# A single buried cysteine acts as a hydrophobic stabilizer of a folding intermediate and transition state in the MATH domain of SPOP

**DOI:** 10.1002/pro.70138

**Published:** 2025-05-14

**Authors:** Livia Pagano, Awa Diop, Valeria Pennacchietti, Mariana Di Felice, Eduarda S. Ventura, Julian Toso, Angelo Toto, Stefano Gianni

**Affiliations:** ^1^ Dipartimento di Scienze Biochimiche “A. Rossi Fanelli” Sapienza Università di Roma Rome Italy; ^2^ Laboratory affiliated to Istituto Pasteur Fondazione Cenci Bolognetti Rome Italy

**Keywords:** cysteine, kinetics, MATH domain, protein folding, site‐directed mutagenesis

## Abstract

Cysteine is a highly conserved amino acid with diverse roles in protein function. Whilst its role in the formation of disulfide bridges is well characterized, the contribution of isolated cysteines in protein folding is by and large unexplored. Here we investigate the impact of cysteine residues on the folding pathway of the MATH domain in the SPOP protein by comparing wild‐type and serine mutants. Through kinetic analyses, we demonstrate that a buried cysteine residue stabilizes both an early folding intermediate and the main transition state. Most notably, such effects are disrupted upon substitution with serine but preserved with alanine. These findings suggest that, in certain structural contexts, cysteine behaves as a hydrophobic rather than a polar residue. Our results challenge the traditional classification of cysteine as a polar amino acid and highlight its unique contributions to protein folding, with implications for protein engineering and structural biology.

## 1 INTRODUCTION

Cysteine is a highly versatile amino acid that, despite its relatively low abundance in many organisms, has been preserved throughout evolution. In fact, together with glycine, proline, and tryptophan, cysteine represents the most evolutionary conserved residue (Marino & Gladyshev, 2010; Marino & Gladyshev, 2011). Such evolutionary conservation underscores the diverse roles of cysteine in proteins spanning from catalysis, regulation, structural stabilization, to cofactor binding (Marino & Gladyshev, 2012). Furthermore, the ability to form disulfide bridges is particularly significant, as these bonds serve as the only natural covalent connections between separate polypeptide strands as well as within a single polypeptide chain, contributing critically to the stability of protein structures (Sevier & Kaiser, 2002).

The chemical and physical classification of Cys has often represented a subject of debate, specifically regarding whether it should be considered hydrophobic or polar (Fersht & Dingwall, 1979; Kyte & Doolittle, 1982; Nagano et al., 1999; Rose, 1978; Sevier & Kaiser, 2002; Taylor, 1986; Wimley & White, 1996). Despite many textbooks shallowly classifying Cys as a polar amino acid, structural analysis appears to suggest that cysteine may behave as a hydrophobic residue, being very frequently buried within the nonpolar core of a proteins. Indeed, an analysis of 15,000 random Protein Data Bank (PDB) structures revealed that isolated cysteines represent the most buried amino acid, followed by Ile, Val, and Ala (Marino & Gladyshev, 2010).

In the context of protein folding, the role of isolated cysteines has been largely overlooked. In fact, while many experimental and computational works have addressed the role of S–S bridges in folding (Johnson et al., 1997; Parrini et al., 2008; Pecher & Arnold, 2009; Sanchez‐Romero et al., 2013; Vogl et al., 1995), primarily highlighting their entropic stabilization of the denatured state, the inability to produce conservative mutations of Cys has generally deterred from including this residue in mutational works (Fersht, 2024; Fersht & Sato, 2004). Furthermore, to avoid undesired multimerization in vitro, cysteine is frequently substituted with serine, based on the assumption that sulfur and oxygen have similar chemical properties (Smertina et al., 2022; Smith et al., 2021; van der Lee et al., 2014; Xia et al., 2015).

Like serine, cysteine can form hydrogen bonds and remains mostly protonated at physiological pH. However, according to the electronegativity scale of Linus Pauling (where oxygen, sulfur, and hydrogen have values of 3.44, 2.58, and 2.2, respectively) (Pauling, 1932), the hydroxyl group forms a polar covalent bond due to the 1.24 electronegativity difference between oxygen and hydrogen, while the smaller electronegativity difference of 0.4 in the thiol group classifies it as a nonpolar covalent bond. Given carbon's electronegativity of 2.55, the SH bond in thiols more closely resembles the CH bond in terms of polarity characteristics, rather than the OH bond in hydroxyl groups. Hence, the behavior of Cys should more closely align with hydrophobic residues—a conclusion that has been, to date, surprisingly, poorly characterized.

To infer the role of cysteine in folding, we scrutinize here the folding pathway of the MATH domain of the SPOP protein (MATH), which contains four isolated Cys, as a case study. We demonstrate that one of these Cys is responsible for the stabilization of both an early folding intermediate and the main transition state, which are highly destabilized when Cys is replaced by serine. Conversely, replacing Cys with alanine does not perturb the stability of these metastable states, providing a clear‐cut indication of the hydrophobic nature of such Cys. Such an effect had escaped previous attempts to characterize the folding of the same domain via mutational analysis (Marsden et al., 2018). As discussed below, this investigation allows providing new insights into the role of cysteine in protein folding and challenges traditional assumptions regarding its polar or nonpolar classification within the protein moiety.

## 2 RESULTS AND DISCUSSION

The kinetic mechanism of folding of the MATH domain has been previously characterized extensively by using a synergy between stopped‐flow kinetics and site‐directed mutagenesis/circular permutation (Marsden et al., 2018). Briefly, it was observed that folding proceeds with the accumulation of a partially folded, highly compact intermediate. Because of the complexity of the folding data, characterization of such an intermediate could not be thoroughly achieved, and the mutational work was analyzed under mildly destabilizing conditions (3 M GdnHCl), where the intermediate is not populated, and focused primarily on the major transition state of folding. All the experiments were performed in the presence of DTT, thus avoiding the formation of undesired inter‐molecular S–S bonds.

**FIGURE 1:**
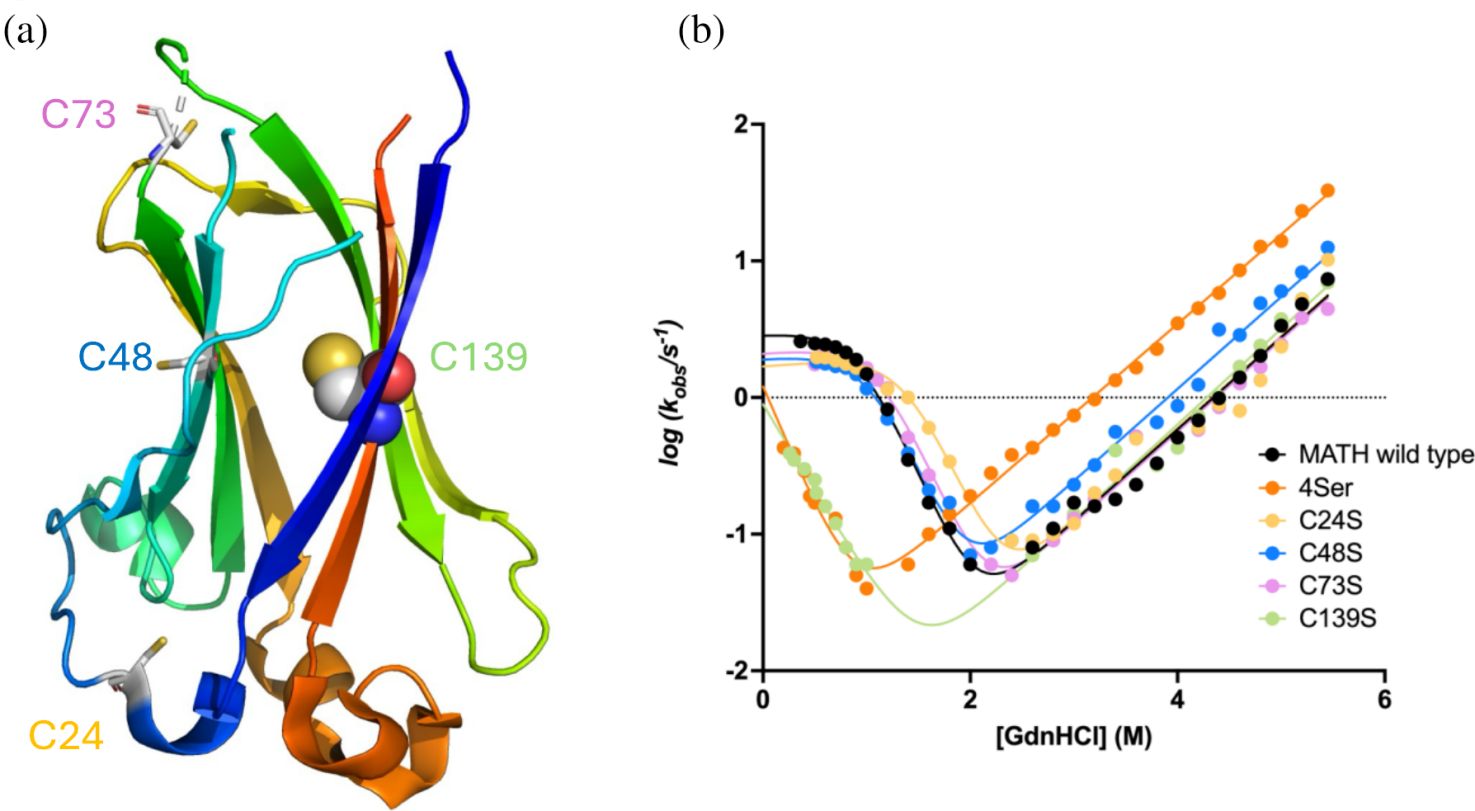
The effect of Cys mutations in the folding of MATH. (a) Three‐dimensional structure of the MATH domain (PDB: 2CR2) with highlighted cysteine residues at positions 24, 48, 73, and 139. As detailed in the text, the buried cysteine in position 139, which is highlighted in spheres, represents the key element in the stabilization of both the folding intermediate and transition state. (b) Kinetic folding experiments of MATH wild type and variants: 4Ser, C24S, C48S, C73S, C139S. Whilst the chevron plots of wild‐type, C24S, C48S, and C73S are consistent with a three state mechanism (as evident from the pronounced curvature at low denaturant concentration), in the case of C139S and 4Ser, the data are consistent with a two‐state V‐shaped scenario. The experiments were performed using a SX‐18 single mixing stopped‐flow device (Applied Photophysics), in buffer 50 mM Hepes pH 7.5 with 10 mM DTT at 37°C, using guanidine hydrochloride as denaturant. Fluorescence emissions were measured with a 360 nm cut‐off glass filter, using an excitation wavelength of 280 nm. At least five individual traces were acquired and then averaged for each concentration of denaturant. Chevron plots were fitted to a three‐state equation, except for the 4Ser variant and C139S, which were fitted with a two‐state equation.

To explore the role of each of the four Cys in the folding of MATH, we produced the respective site‐directed variants in which each of them was replaced with Ser, namely C24S, C48S, C73S, and C139S. Additionally, a quadruple variant where all Cys were mutated to Ser, named 4Ser, was also expressed and purified. The structure of MATH (Usher et al., 2021), highlighting the four cysteine residues, is reported in Figure 1a.

In analogy to the previous work by Clarke and co‐workers (Marsden et al., 2018), the folding and unfolding kinetics of MATH and its variants were investigated by stopped‐flow, triggered by an 11‐fold dilution of the denatured or the native protein into the appropriate buffer, and all the experiments were performed in the presence of DTT. In all cases, folding and unfolding time courses were both fitted satisfactorily to a single exponential decay at any final denaturant concentration. A semilogarithmic plot of the folding and unfolding rate constants versus denaturant concentration (chevron plot) of wild‐type MATH, C24S, C48S, C73S, and C139S and 4Ser is reported in Figure 1b. Interestingly, whereas the chevron plots of wt, C24S, C48S, and C73S display a pronounced curvature (roll‐over) in the refolding branches and essentially unaltered rate constants, in the case of C139S, folding is remarkably decelerated and refolding appears linear. Remarkably, the folding arm of 4Ser is very similar to C139S, indicating that the perturbation of folding kinetics may be univocally assigned to the mutation of Cys139 to Ser.

The presence of curvatures in chevron plots represents a typical signature of transient intermediates (Gautier et al., 2020; Gianni et al., 2007; Matouschek et al., 1990; Parker et al., 1995; Travaglini‐Allocatelli et al., 2003). Hence, the clear‐cut loss of curvature observed in the case of C139S and 4Ser variants clearly indicates that Cys139 is critical in the structural organization of such metastable state. Analogously, the remarkable decrease of the folding rate constant associated to the major transition state by nearly 2 order of magnitudes indicates that Cys139 also stabilizes the major folding transition state. This conclusion is in line with what previously suggested by Clarke and co‐workers, who suggested the folding transition state of MATH to be primarily stabilized by the interaction between the N‐ and C‐terminal strands (Marsden et al., 2018), with Cys139 being located in the latter (Figure 1a). We note, however, despite being an apparent “conservative” mutation, C139S displays the most remarkable change in folding rate constant, compared to all the 35 variants previously characterized, indicating that this residue represents the key element in the stabilization of both the intermediate and transition state.

On the light of the results highlighted above, it is possible to conclude that the buried Cys139 has a key role in stabilizing the folding intermediate and transition state. To investigate if such stabilization might be ascribed to the hydrophobic nature of buried cysteine, we conducted two key experiments. First, we compared the folding behavior of the wild‐type protein and the C139S mutant under neutral and alkaline pH conditions. Second, we substituted C139 with alanine to assess its impact on protein refolding.

**FIGURE 2:**
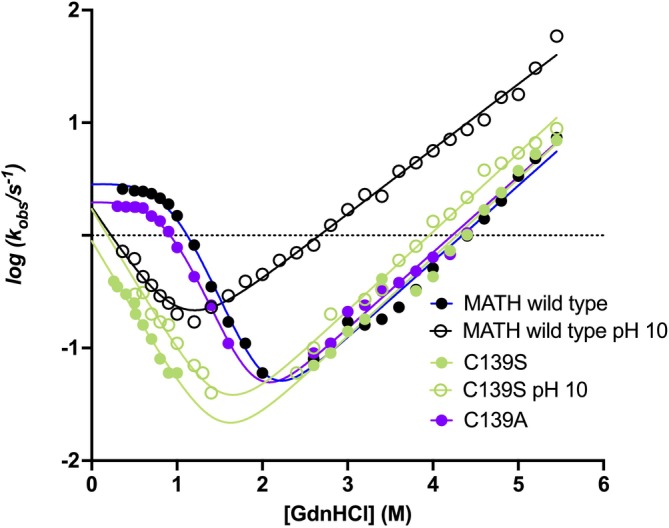
Comparison of kinetic folding experiments of MATH wild type (black circle) and variants C139S (green circle), C139A (violet circle) performed in buffer 50 mM Hepes pH 7.5 and in buffer 50 mM CHES pH 10 both supplemented with 10 mM DTT at 37°C. Chevron plots of MATH wild type at pH 10 and the mutant C139S, in both experimental conditions, were fitted to a two‐state equation, while, MATH wild type and C139A mutant at pH 7.5 were fitted to a three‐state equation.

Amino acids, like other molecules, contain ionizable groups (e.g., –OH, –NH₂, –SH) that can alter their protonation state depending on the pH of the solution. By adjusting the pH above or below the pKa of a specific side chain, it is therefore possible to modulate its protonation state. Given that the pKa of cysteine is approximately 9.5, at pH 10, the thiol group is likely deprotonated, forming a thiolate ion. The thiolate form exhibits significantly more polar characteristics, similar to serine, and cannot act as a hydrophobic side chain. The chevron plot of MATH wild type measured at pH 10 is reported in Figure 2. Remarkably, the chevron plot at pH 10 displays a clear destabilization of the intermediate and appears to revert to a two‐state folding mechanism, with the observed refolding arm being very similar to those of C139S and 4S. Moreover, the alkaline pH induced perturbation of folding is completely abolished in the case of C139S, which returns nearly identical chevron at alkaline and neutral pH (Figure 2). Hence, it appears that deprotonation of Cys139 disrupts its ability to stabilize the folding intermediate by acting as a hydrophobic residue and when Cys139 is replaced by serine, which is inherently polar, the folding mechanism remains unaffected regardless of pH.

As anticipated above, to further prove the hydrophobic nature of Cys139 within the MATH structure, we substituted it with alanine and subjected it to folding and unfolding experiments. To our delight, the variant C139A displays nearly identical folding and unfolding kinetics if compared to the wild type protein (Figure 2). This finding provides unequivocal evidence that Cys139 behaves as a hydrophobic residue within the MATH structure. Of additional note, the same variant was also expressed in reference (Marsden et al., 2018) and returned nearly identical results as those reported in this work, yet failure to investigate in detail the hydrophobic nature of C139 has previously hidden its role in the stabilization of the folding intermediate and transition state.

The results described in this work are of special interest in at least two ways. First, we show that buried cysteines might have a critical role in stabilizing folding intermediates and transition states. Whilst many studies have previously focused on the role of S–S bridges in sculpting the entropy of the denatured states (Johnson et al., 1997; Parrini et al., 2008; Pecher & Arnold, 2009; Sanchez‐Romero et al., 2013; Vogl et al., 1995), the contribution of isolated cysteines to folding stability has remained largely unexplored. Our findings demonstrate that even in the absence of disulfide bonds, buried cysteines can exert a significant stabilizing effect on transient folding intermediates, emphasizing their functional importance beyond redox chemistry. Second, our results provide an additional challenge to the traditional classification of cysteine as a polar amino acid and advocate for a more nuanced understanding of its behavior in proteins. We highlight the limitations of the common assumption that cysteine can be straightforwardly substituted by serine in a conservative manner and underscore the unique structural and functional contributions of cysteines. These findings could have broader implications for protein engineering and folding studies, particularly in the design of cysteine‐containing proteins.

## 3 MATERIALS AND METHODS

### 3.1 Protein expression and purification

The construct encoding MATH wildtype (purchased from Eurofins Genomics) and all Serine (4Ser) variants were subcloned in a pHTP1 vector, previously described in https://www.nzytech.com/en/mb282-nzyeasy-cloning-expression-kit-i/. Four variants C24S, C48S, C73S, and C139S, corresponding to the substitution of a single cysteine residue to Serine, as well as the C139A variant, were each generated using the QuikChange Lightning Mutagenesis Kit (Agilent technologies, Inc., Santa Clara, CA, USA) following manufacturer instructions. Primers were purchased from Eurofins Genomics, and the sequences of all constructs were confirmed by DNA sequencing. All constructs encoding MATH variants were subcloned in a pHTP1 plasmid vector and then transformed in Escherichia coli BL21 (DE3) cells. Bacterial cells were grown in LB medium, containing 30 μg/mL of kanamycin, at 37°C until OD600 = 0.7–0.8, and then protein expression was induced with 1 mM IPTG. After induction, cells were grown at 25°C overnight and then collected using centrifugation (10 min, 5000 rpm). To purify the His‐tagged protein, the pellet was resuspended in buffer made of 50 mM Tris–HCl, 300 mM NaCl, and 10 mM Imidazole, pH 7.5, and with the addition of an antiprotease tablet (cOmplete, EDTA‐free, Roche Diagnostics GmbH, Mannheim, Germany), then sonicated and centrifuged. The soluble fraction from bacterial cell lysate was loaded onto a nickel‐charged HisTrap Chelating HP (GE Healthcare Bio‐Sciences AB, Uppsala, Sweden) column equilibrated with the same buffer. Each protein variant was then eluted with a gradient from 0 to 1 M imidazole by using an ÄKTA‐prime system. Fractions containing the protein were collected, and the buffer was exchanged to 50 mM Tris–HCl and 300 mM NaCl, pH 7.5, using HiTrap desalting columns (GE Healthcare). The purity of all protein variants was assessed through SDS‐PAGE.

### 3.2 Stopped flow (un)folding kinetics experiments

(Un)folding kinetics experiments were performed on an Applied Photophysics Pi‐star 180 stopped‐flow apparatus, monitoring the change of fluorescence emission, exciting the sample at 280 nm, and recording the fluorescence emission by using a 320 nm cut‐off glass filter. The experiments were performed at 298 K, by using buffers at neutral (Tris–HCl pH 7.5 buffer) and alkaline (CHES buffer pH 10) pH with Guanidinium chloride (GdnCl) as a denaturant agent. The final protein concentration was typically 2 μM. For each denaturant concentration, at least five individual traces were averaged. All the observed time courses were analyzed with single exponential phases using the fitting procedure provided by the Applied Photophysics software.

## AUTHOR CONTRIBUTIONS

L.P., A.D., V.P., M. D. F., E. S. V. J.T. performed experimental work and analyzed the data; A.T. and S.G. supervised the work, analyzed data, and gained funding support. L.P. and S.G. wrote the first version of the manuscript, which was then edited by all authors. All authors have given approval to the final version of the manuscript.

## FUNDING INFORMATION

This work was partly supported by grants from European Union's MSCA Doctoral Networks under the Grant Agreement IDPro No 101119633 (to S.G.), by grants from Sapienza University of Rome (RG12017297FA7223, RG1231888A88129D to S.G., RG124190E53A81B8, RM12218148DA1933 to A.T.), the Associazione Italiana per la Ricerca sul Cancro (Individual Grant—IG 24551 to S.G.), the Istituto Pasteur Italia (“Research Program 2022 to 2023 Under 45 Call 2020” to A.T.), the Italian MUR‐PRIN 2022 grant N. 2022JY3PMB to A.T. We acknowledge co‐funding from Next Generation EU, in the context of the National Recovery and Resilience Plan, and the Investment PE8—Project Age‐It: “Ageing Well in an Ageing Society”. This resource was co‐financed by the Next Generation EU [DM 1557 11 October 2022]. The views and opinions expressed are only those of the authors and do not necessarily reflect those of the European Union or the European Commission. Neither the European Union nor the European Commission can be held responsible for them.

## Data Availability

The data that support the findings of this study are available from the corresponding author upon reasonable request.
